# A case of COVID-19 in a patient with a univentricular heart post total cavopulmonary connection (Fontan) surgery

**DOI:** 10.1017/S1047951120001882

**Published:** 2020-06-16

**Authors:** Niall Linnane, Des W. Cox, Adam James

**Affiliations:** 1 Department of Cardiology and Cardiac Surgery, Children’s Health Ireland, Crumlin, Dublin, Ireland; 2 Department of Respiratory Medicine, Children’s Health Ireland, Crumlin, Dublin, Ireland; 3 School of Medicine, University College Dublin, Dublin, Ireland

**Keywords:** COVID-19, Fontan, CHD, univentricular circulation

## Abstract

Coronavirus disease 2019 (COVID-19) has caused a global pandemic which has affected patients and healthcare systems around the world. Patients with underlying health conditions seem to be more severely affected. There are limited reports of patients with univentricular circulations and COVID 19; thus, we report a case of COVID-19 in a patient with a univentricular circulation.

Coronavirus disease 2019 (COVID-19), which is caused by severe acute respiratory syndrome virus 2 (SARS COV-2), has caused a global pandemic since being first described in Wuhan, China, in December, 2019.^1^ Currently, according to the World Health Organization (9 June, 2020), there are 7,039,918 confirmed cases with 404,396 deaths. A large population-based registry in the United States estimated that by April, 6, 176,190 children were infected and 74 were admitted to ICU.^2^ This highlights that most children appear to have a mild course. Among 345 COVID-19 paediatric cases, whose data on underlying conditions were available, the Centres for Disease Control and Prevention reported that the most common underlying conditions were chronic lung disease (11.6%), cardiovascular disease (7.2%), and immunosuppression (2.9%). This report has limited data with only 295 cases having information both on hospitalisation status and underlying medical conditions, but showed 77% of admitted children and 100% children admitted to ICU had underlying health conditions.

Whereas COVID-19 is primarily a respiratory infection, it has important systemic effects. There is limited evidence of the effect of COVID-19 on patients living with underlying CHD.^3^ Within children with CHD, patients with univentricular circulations are considered at the severe end of the spectrum, with many paediatric cardiology centres referring to them as high risk in the current COVID-19 pandemic.

We report to the best of our knowledge the first case of COVID-19 in a patient who has a univentricular circulation post Fontan surgery.

## Case

A 10-year-old boy with a background of double inlet left ventricle, pulmonary atresia, atrial septal defect, and a right aortic arch originally presented to cardiology at 8 months of life due to chronic respiratory issues and cyanosis. He proceeded to have a bidirectional Glenn procedure and completed a total cavopulmonary connection via an extracardiac fenestrated Fontan surgery at 3 years and 10 months.

His last outpatient visit was 1 month prior to developing COVID-19, at which point, his oxygen saturations were 98%, heart rate 83 bpm, and blood pressure (BP) 124/60. His weight was 78.23 kg, height was 150.2 cm^2^, and BMI 34.7 kg/m^2^ (>99.6th centile).

He was initially presented to his local hospital with symptoms of fever, red eyes, lethargy, and mild cough. His initial presentation was prompted as his parents were positive for SARS-COV-2 and his contact tracing swab was positive. He was well with normal saturations and was discharged home. He re-presented 4 days later with increased work of breathing, worsening cough, and persistent fever. He had an oxygen requirement (0·5 L/min) at this stage to maintain oxygen saturations >90%.

**Figure 1.**
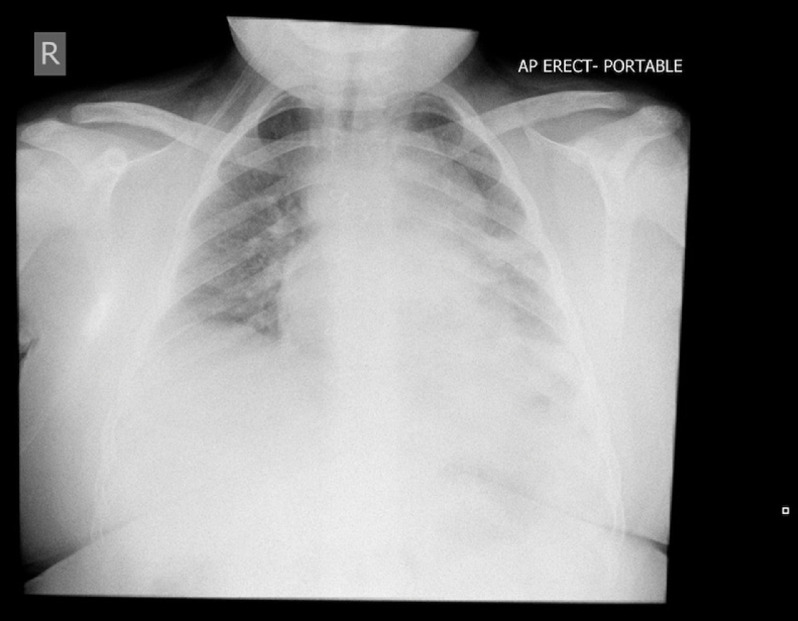
Chest X-ray day 7.

On day 7 of his illness, he deteriorated requiring an increase in his oxygen requirement to 1 L/min, and his chest x-ray showed significant left mid and lower zone consolidation (Fig 1). He was commenced on intravenous cephalosporin and oral macrolide antibiotics. There had been ongoing liaison since his initial presentation with paediatric cardiology and respiratory specialists, and it was decided with the deterioration to transfer to a tertiary paediatric hospital.

**Figure 2.**
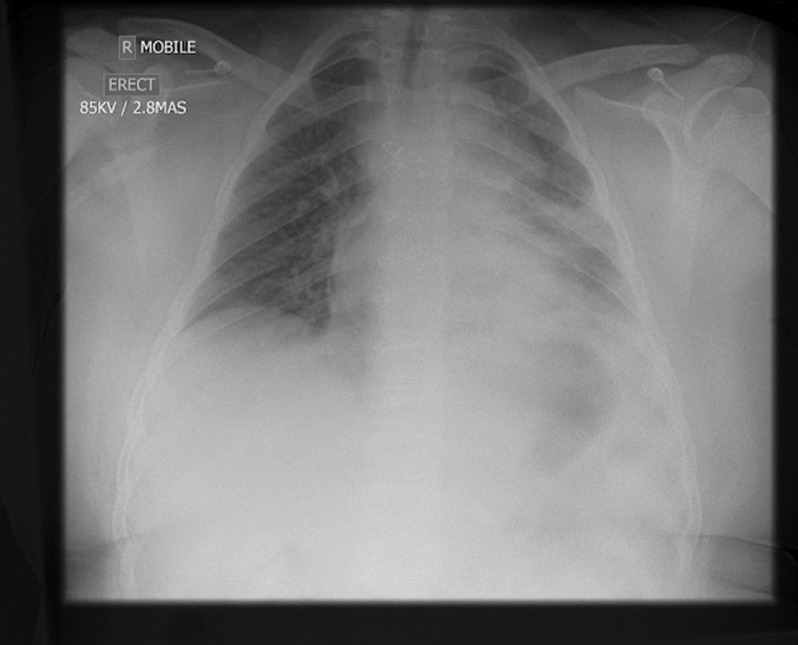
Chest X-ray day 9.

His oxygen requirement increased to a peak of 3 L/min from day 8 to 10 of illness. His chest x-ray, however, on day 9 was unchanged (Fig 2). His full blood count showed haemoglobin 125 g/L, platelets 144 × 10^9^/L, and total white cell count 2.4 × 10^9^/L with neutrophils 1.67 × 10^9^/L and lymphocytes 0.56 × 10^9^/L. His C-reactive protein was 13. He had a normal renal profile and normal liver profile. Further escalation of ventilatory support was not required, and he remained at ward level care for the duration of his admission. Intravenous antibiotics were stopped after 7 days, and no further medicines were prescribed. He was transferred back to his local hospital on day 14 of his illness self-ventilating in room air prior to his discharge home.

## Discussion

A number of studies have reviewed the symptoms and signs present in adults, but few have looked specifically at children.^1^ There have been case reports of COVID-19 in specific groups, but a large systematic review failed to identify any studies that quantified the prevalence of comorbidities in children diagnosed with COVID-19.^1^ Children have represented some 2% of diagnosed cases in China, 1·2% of cases in Italy, and 5% in the United States. A large systematic review showed that 90% of children have mild or moderate disease, 5.2% have severe, and 0.6% critical disease. Severe disease is defined as dyspnoea, cyanosis, and oxygen saturations <92%. Critical disease required respiratory failure, shock, and signs of multi-organ failure.^1^ Thus, our patient was diagnosed with severe disease.

There have been reports of an inflammatory syndrome occurring in children infected with SARS-COV-2 which seems to be at the severe end of the Kawasaki disease spectrum and can lead to rapid deterioration and ICU admission.^4^ These have been described in previously well children and not in those with univentricular circulations. Our case presents with primarily respiratory symptoms and no evidence of cardiovascular compromise.

In a recent report by Ahluwalia et al of an adult patient with tricuspid atresia and status post Fontan palliation presenting with COVID-19,^5^ it was emphasised that haemoglobin can aid in differentiating acute hypoxia from chronic hypoxia. Similarly, our patient had both normal oxygen saturations in the clinic and a normal haemoglobin concentration on admission demonstrating that there was acute hypoxia secondary to COVID-19.

Fontan physiology exerts a considerable impact on the pulmonary circulation, characterised by increased pulmonary vascular impedance. The lack of a pulsatile blood flow has major effects on endothelial function, vascular recruitment, and lung vessel growth, which in turn influence pulmonary vascular resistance.^6^ In Fontan physiology, blood ﬂow through the pulmonary arteries is non-pulsatile and largely driven by negative intrathoracic pressure and systemic BP. When cardiac output increases in a normal individual, pulmonary vessels which are normally under-perfused are recruited, thereby responding to the increase in ﬂow by decreasing pulmonary vascular resistance.^6^ With the absence of pulsatile ﬂow, recruitment is reduced, potentially resulting in increased pulmonary vascular resistance.^6^

This in turn makes patients with Fontan physiology high risk for requiring hospitalisation with acute respiratory infections including COVID-19.

## Conclusion

This case report highlights that despite cardiovascular disease being an important risk factor for morbidity and mortality in adult patients with COVID-19, and the perceived high risk of patients with univentricular circulation, as of yet cardiovascular disease has not been shown to be an important risk factor for children with COVID-19. Our patient was diagnosed as having severe COVID-19 on this presentation, and despite his univentricular circulation, he was managed with conservative measures and did not require escalation to ICU level care.
